# NMDA attenuates the neurovascular response to hypercapnia in the neonatal cerebral cortex

**DOI:** 10.1038/s41598-019-55468-1

**Published:** 2019-12-11

**Authors:** Gábor Remzső, János Németh, Valéria Tóth-Szűki, Viktória Varga, Viktória Kovács, Ferenc Domoki

**Affiliations:** Department of Physiology, Faculty of Medicine, University of Szeged, Szeged, Hungary

**Keywords:** Neuro-vascular interactions, Blood flow, Translational research

## Abstract

Cortical spreading depolarization (SD) involves activation of NMDA receptors and elicit neurovascular unit dysfunction. NMDA cannot trigger SD in newborns, thus its effect on neurovascular function is not confounded by other aspects of SD. The present study investigated if NMDA affected hypercapnia-induced microvascular and electrophysiological responses in the cerebral cortex of newborn pigs. Anesthetized piglets were fitted with cranial windows over the parietal cortex to study hemodynamic and electrophysiological responses to graded hypercapnia before/after topically applied NMDA assessed with laser-speckle contrast imaging and recording of local field potentials (LFP)/neuronal firing, respectively. NMDA increased cortical blood flow (CoBF), suppressed LFP power in most frequency bands but evoked a 2.5 Hz δ oscillation. The CoBF response to hypercapnia was abolished after NMDA and the hypercapnia-induced biphasic changes in δ and θ LFP power were also altered. MK-801 prevented NMDA-induced increases in CoBF and the attenuation of microvascular reactivity to hypercapnia. The neuronal nitric oxide synthase (nNOS) inhibitor (N-(4 S)-4-amino-5-[aminoethyl]aminopentyl-N′-nitroguanidin) also significantly preserved the CoBF response to hypercapnia after NMDA, although it didn’t reduce NMDA-induced increases in CoBF. In conclusion, excess activation of NMDA receptors alone can elicit SD-like neurovascular unit dysfunction involving nNOS activity.

## Introduction

Spreading depolarizations (SDs) are repeatable, slowly (3–4 mm/min) propagating waves of almost complete neuronal and glial depolarization at the wavefront with simultaneous depression of cortical electrical activity (cortical spreading depression). These electrophysiological changes are accompanied by transient elevations in interstitial H^+^, K^+^, glutamate levels and by a characteristic triphasic, in intact brains dominantly hyperemic microvascular response, the latter presumably helping to meet the increased metabolic demand necessary for the recovery of transmembrane ion gradients. In various neurological disorders, such as ischemic stroke or traumatic brain injury, SDs have been shown to play critical roles in the development of cerebrocortical lesions^1^, therefore it is of great interest to identify the mechanisms by which SDs may contribute directly or indirectly to cortical neuronal injury.

One of the important injury mechanisms of SDs can be the induction of neurovascular unit dysfunction. SDs have been long reported to alter cortical microvascular reactivity, for instance, a single SD can abolish the normal microvascular reactivity to hypercapnia in virtually all species studied including cats^2^, rats^3^ and mice^4^. However, the mechanism of SD-induced microvascular dysfunction remains unclear.

N-methyl-d-aspartate (NMDA), the prototypical selective activator of the NMDA subtype of ionotropic glutamate receptors, has been shown to trigger SDs when applied topically onto the surface of the cerebral cortex in adult mice^5^ and rats^6^. In addition, functional NMDA receptors are clearly required for triggering SDs with other stimuli and also for SD propagation^7,8^. Interestingly, SDs cannot be elicited in neonates, despite expressing functional NMDA receptors. In the newborn pig, local application of NMDA has been shown to dilate pial arterioles and to increase cortical blood flow in a dose-dependent fashion without confounding SDs^6^.

In the present study, we set out to investigate the effect of topical cortical application of NMDA on the cerebrocortical microvascular response to graded hypercapnia in a neonatal piglet model using laser-speckle contrast imaging (LSCI). Using a neonatal model, we wished to assess only one aspect of SD – the NMDA receptor activation- on neurovascular unit function without the confounding features of an actual SD triggered by NMDA in adult brains. As we found a significant attenuation of CO_2_-reactivity after NMDA application similar to those previously observed in adult animal models following SDs, we used the NMDA-receptor inhibitor MK-801 and the selective neuronal nitric oxide synthase (nNOS) inhibitor (N-(4 S)-4-amino-5-[aminoethyl]aminopentyl-N′-nitroguanidin; AAAN) to pharmacologically characterize this effect. Furthermore, we sought to determine and describe for the first time the cortical layer-specific changes in electrical activity triggered by local NMDA application in this neonatal model recorded with multi-channel microelectrodes.

## Materials and Methods

### Animals and surgery

Newborn (<24 h old) male Landrace piglets (n = 31, body weight: 1.5–2 kg) were obtained from a local company (Pigmark Ltd., Co., Szeged, Hungary). The experimental procedures were approved by the National Ethical Committee on Animal Experiments (ÁTET, I.74–7/2015), and then the necessary permit to obtain the animals was issued by the National Food Chain Safety and Animal Health Directorate of Csongrád county, Hungary (permit nr: XIV./1414/2015). The procedures were performed according to the guidelines of the Scientific Committee of Animal Experimentation of the Hungarian Academy of Sciences (updated Law and Regulations on Animal Protection: 40/2013. (II. 14.) Gov. of Hungary), following the EU Directive 2010/63/EU on the protection of animals used for scientific purposes and reported in compliance with the ARRIVE guidelines.

The animals were restrained and anesthetized with intraperitoneal sodium thiopental injection (45 mg/kg; Sandoz, Kundl, Austria). The animals were placed on a servo-controlled heating pad (Blanketrol III, Cincinnati SUB-zero, Cincinnati, Ohio, USA), keeping their core temperature in the physiological range (38.5 ± 0.5 °C). The piglets were intubated through a tracheostomy then mechanically ventilated with humidified medical air occasionally supplemented with oxygen (FiO_2_: 0.21–0.25) with the following ventilation parameters: respiration rate (RR): 30–35/min; peak inspiratory pressure (PIP): 12–14 cmH_2_O. A catheter was inserted into the right femoral vein under aseptic conditions and anesthesia/analgesia was switched to intravenous morphine (100 μg/kg bolus then 10 μg/kg/h; Teva, Petach Tikva, Israel) and midazolam (250 μg/kg bolus then 250 μg/kg/h; Torrex Pharma, Vienna, Austria) as used previously^9,10^ along with supportive fluid therapy (0.45% NaCl, 5% glucose; 3 ml/kg/h). A second catheter was inserted into the right carotid artery for taking blood samples, monitoring the mean arterial blood pressure (MABP) and heart rate (HR). As shown previously, unilateral carotid artery occlusion does not affect cerebral blood flow (CBF) and preferable to catheterization of the femoral artery causing very severe hindlimb ischemic damage^11^. Blood samples (300 μl) were analyzed for pH, gases, electrolytes and metabolites with an epoc^®^ Blood Analysis System (Epocal Inc., Ottawa, Canada). We monitored the peripheral saturation (SpO_2_) using pulse oximetry.

After instrumentation, the heads of the animals were fixed into a stainless steel stereotactic frame (RWD Life Science, Shenzhen, Guangdong Province, China). For the LSCI studies, we implanted a stainless steel closed cranial window (d = 1.8 cm) over the parietal cortex which was sealed with bone wax and cemented with dental acrylic^12^. For the electrophysiology studies, we obtained an open cranial window over the left parietal bone for electrode insertion and we also drilled two holes into the frontal bone positioning the reference and ground electrodes, respectively. The dura mater was carefully removed avoiding the blood vessels. If necessary, the smaller veins were cauterized. The location of the cranial window and the electrode insertion point was determined by stereotactic reference points (window d = 0.8 cm; measured from Bregma: anterior-posterior (AP) axis:-1.2-(−1.4) cm, medial-lateral (ML) axis: 1.1–1.3 cm). The subarachnoidal space was filled with warmed (37 °C) artificial cerebrospinal fluid (aCSF) containing 0.22 g/l KCl, 0.132 g/l MgCl_2_, 0.221 g/l CaCl_2_, 7.71 g/l NaCl, 0.402 g/l urea, 0.665 g/l dextrose and 2.066 g/l NaHCO_3_, and was equilibrated with a gas mixture containing 6.3% O_2_, 6.2% CO_2_, and 87.5% N_2_, respectively. At the end of the experiments the animals were euthanized with an overdose of pentobarbital sodium (300 mg, Release; Wirtschaftsgenossenschaft deutscher Tierärzte eG, Garbsen, Germany).

### Experimental protocol

**Figure 1:**
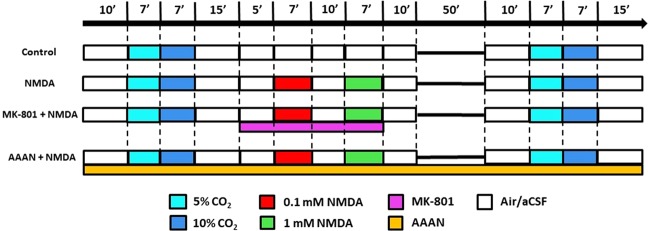
Experimental groups and protocol. The animals fitted with closed cranial windows for laser-speckle contrast imaging and analysis were divided into 4 groups. In the control group, (Group 1, n = 6) graded hypercapnia was induced with 5-10% CO_2_ that was repeated after 1 h. In Group 2 (n = 7), increasing concentrations (0.1–1 mM) of NMDA dissolved in artificial cerebrospinal fluid (aCSF) were applied topically to the cortex between the two hypercapnic stimuli. In Group 3 (n = 4), the protocol was the same as in the second, only the NMDA-receptor inhibitor MK-801 (0.1 mM dissolved in aCSF) was pre- and co-applied with NMDA. In Group 4 (n = 7), the protocol was also similar to Group two except that the animals were intravenously treated with the selective neuronal nitric oxide synthase inhibitor N-(4 S)-4-amino-5-[aminoethyl]aminopentyl-N′-nitroguanidin (AAAN; 0.4 mg/kg) before the first graded hypercapnia. An additional group of animals (n = 7) fitted with open cranial windows served for the electrophysiology studies using the same protocol as for the NMDA-treated second group.

LSCI and electrophysiology studies were performed in separate animals. For the LSCI measurements the animals were divided into 4 groups (Fig. 1). Randomization was performed by coin flip between Group 1 and 2, or 3 and 4, respectively. In each group after obtaining baseline, the microvascular response to graded hypercapnia induced by mechanical ventilation with 5% and 10% CO_2_ for 7 min for each concentration was recorded. In Group 1 (n = 6), the graded hypercapnia was repeated after recovery time matching the NMDA-treated groups. In Group 2–4, after the first graded hypercapnia increasing concentrations (0.1 and 1 mM) of N-methyl-d-aspartate (NMDA; Sigma Aldrich, St. Louis, MO, US) dissolved in aCSF were applied topically on the cortical surface, each NMDA stimulus lasted for 7 min (Fig. 1). Between and after the NMDA applications, the cranial window was washed with aCSF for 10 min to allow CoBF and the electrical activity to return to baseline. The second graded hypercapnia was elicited 1 h after completion of the NMDA application. In Group 2 (n = 7), the effect of NMDA was studied, in Group 3 (n = 4), the cortex was locally pre-treated with the NMDA receptor antagonist MK-801 (0.1 mM dissolved in aCSF; Research Biochemicals International, Natick, MA, US) and the NMDA was also co-applied with 0.1 mM MK-801. Finally in Group 4 (n = 7), before the start of the LSCI protocol the animals were given a selective neuronal nitric oxide synthase (nNOS) inhibitor (N-(4 S)-4-amino-5-[aminoethyl]aminopentyl-N′-nitroguanidin; AAAN; Santa Cruz Biotechnology, Dallas, TX, US; dissolved in saline, 0.4 mg/kg iv)^13^. In each group, LSCI was recorded for additional 5 min after euthanasia to determine the biological zero.

Electrophysiology studies were performed with the same protocol as in Group 2 (n = 7). We tested if the cerebrocortical response to NMDA markedly differed in the absence of preceding graded hypercapnia in two additional animals, but we did not observe any differences (data not shown).

### LSCI measurements and analysis

The brain was illuminated with near infrared polarized light (λ = 808 nm, 200 mW) with a laser diode (DL-8141-002 SANYO Electric Co., Japan). The images were recorded with a monochrome camera (PL-B741F; PixeLINK® Ottawa, Canada; 1280 × 1024 pixels) which is using a polarizer and a color filter. The raw speckle images were sampled at 1 Hz, 1 ms exposure time with 1 frame/s rate during all the vascular stimuli.

The LSCI analysis was performed offline in LabVIEW (National Instruments Co., Austin, TX, USA). The contrast maps were calculated from the raw speckle images using a 5 × 5 pixel window. In each animal we selected 4 parenchymal region of interests (ROIs; 5 × 5 pixels ~1200 µm^2^) over the cortical parenchyma avoiding pial vessels. The τ correlations were calculated using Eq. (1), where K(T) is each ROIs’ speckle contrast, β is the coherence factor and T is the exposure time.

1 $$K(T)=\sqrt{{\rm{\beta }}}{\{\frac{{{\rm{\tau }}}^{2}}{2{T}^{2}}[exp(\frac{-2T}{{\rm{\tau }}})-1+\frac{2T}{{\rm{\tau }}}]\}}^{\frac{1}{2}}$$

For each image, the calculated 1/τ values were normalized and expressed as percentages of the baseline, and values from all 4 ROIs were averaged in each animal.

Pial arteriolar diameters were determined at selected time points where peak changes in CoBF to the applied stimuli were detected. To obtain better resolution, 30 images were averaged then the Otsu filtering method was applied to reduce noise. In each experiment, four arterioles were selected and the internal diameter of the arterioles were determined using edge detection and Euclidean distance measurement in MATLAB (Mathworks Inc., Natick, US)^14^. The arteriolar diameter data were normalized and expressed as percentages of the baseline, and values from all 4 arterioles were averaged in each animal.

### Neurophysiological recordings

Electrophysiological recordings were taken with 16-channel, acute single shank planar silicone electrodes (l = 10 mm, base width = 50 µm) with 177 µm^2^ recording sites spaced 100 µm apart (A1x16-10mm-100-177-A16; Neuronexus Technologies. AnnArbor, MI, US, http://neuronexus.com/electrode-array/a1x16-10mm-100-177/). Data acquisition was performed with RHD2000 Evaluation System. The recorded signals were preamplified with a 16-channel headstage/amplifier board (RHD2132 amplifier board, Intan Technologies, Los Angeles, US) under Faraday-cage then the signals were sent through an interface cable to the interface board (RHD2000 USB interface board, Intan Technologies, Los Angeles, US). All recorded data were sampled at 20 kHz, the broad band signals were filtered with a 1-9000 Hz bandpass filter and a notch filter was also applied to eliminate the 50 Hz electrical noise. All data were analyzed off-line in MATLAB environment with implemented toolboxes (Chronux, http://chronux.org/; FMAtoolbox, http://fmatoolbox.sourceforge.net) and custom written scripts.

### LFP spectral analysis

The recorded broad band signals (20 kHz) were downsampled to 1250 Hz and filtered with an infinite impulse response (IIR) 4^th^ order Butterworth filter to generate the local field potential (LFP) and to eliminate spiking activity. After LFP generation, we decomposed the signal into the physiological frequency ranges - delta (1–4 Hz), theta (4–8 Hz), alpha (8–13 Hz), beta (13–30 Hz) – to calculate the power spectral density (PSD). We applied a 30 s window on the signals which moves onward with 1 s steps calculating the Fast Fourier Transform (FFT) of the signals using a Gaussian window. The PSDs were determined for each channels, frequency bands, conditions and animals. The calculated PSDs were summed for each frequency bands. All the PSDs were averaged and normalized to the baseline activity.

Addressing the ‘inverse problem’ of LFP, we computed the second spatial derivative, the current source density (CSD) to reveal how the different sources contribute to the mixed signal. We explored and segmented the data into 2.5 s epochs using Neuroscope^15^. We used the standard CSD method for the computation. All CSDs were calculated with the FMAtoolbox’s built-in function and the spectra were averaged.

### Spike sorting and unit classification

Spike sorting was done with the Klusta package (https://github.com/kwikteam/klusta) that performs automatic spike sorting and clustering. Single units were detected from digitally 1–5000 Hz high-pass filtered LFP using a threshold crossing-based algorithm (Spikedetekt2; https://github.com/klusta-team/spikedetekt2). The detected spikes were automatically clustered using masked Expectation-Maximization (EM) algorithm for Gaussian mixtures which is implemented into KlustaKwik2^16^ (https://github.com/klusta-team/klustakwik2/). Clustering was followed by manual adjustment of the clusters using phy KwikGUI software (https://github.com/kwikteam/phy) which is an improved version of KlustaViewa^17^. The noise, as well as multi-unit and poor quality clusters were discarded from the analysis. The putative interneurons and pyramidal cells were identified by their waveform characteristics and autocorrelograms (ACG)^18–20^ with the further examination of their cross-correlograms (CCG) to reveal the monosynaptic interactions with other single units^19,21^.

### Statistical analysis

All the LSCI statistical analysis were performed in IBM SPSS Statistics 22, using two-way ANOVA with repeated measures, followed by Tukey’s *post hoc* test. All results show mean ± SD, respective to the baseline. p < 0.05* was considered as significant.

The electrophysiological statistical analysis was performed with IBM SPSS Statistics 22. We performed one-way ANOVA with repeated measures, followed by Bonferroni *post hoc* test. All results show mean ± SD, respective to the baseline. p < 0.05* and p < 0.01** were considered as significant. For the Z-score computation we used MATLAB’s statistics toolbox. Relative PSD changes were determined as significant above/below Z ≥ ± 2* and Z ≥ ± 4** with the further examination of the ANOVA results (p < 0.05*, p < 0.01**).

## Results

### Effects of graded hypercapnia on physiological parameters

Ventilation with 5–10% CO_2_ resulted in graded hypercapnia that was similar in all experimental groups both for LSCI and for electrophysiology experiments (Table 1). Graded elevations in arterial pCO_2_ were accompanied by the expected development of marked respiratory acidosis and a slight increase in plasma HCO_3_^−^ levels, however, arterial pO_2_, blood oxygen saturation, MABP and HR were all maintained during graded hypercapnia. The stimulus was highly repeatable, repeated ventilation with 5-10% CO_2_ resulted in virtually identical changes in blood gases compared to the first application (Table 1).

**Table 1:** Arterial pCO_2_, pO_2_, pH, HCO_3_^−^, base excess BE(b), oxygen saturation (SpO_2_), mean arterial blood pressure (MABP) and heart rate (HR) values during the 1^st^ and 2^nd^ stimulation with graded hypercapnia (mean ± SD) (n = 31).

	1^st^ stimulus			2^nd^ stimulus		
	Baseline	5% CO_2_	10% CO_2_	Baseline	5% CO_2_	10% CO_2_
pCO_2_ (mmHg)	39 ± 6	63 ± 11	91 ± 14	42 ± 5	69 ± 7	99 ± 9
pO_2_ (mmHg)	69 ± 9	79 ± 21	82 ± 26	67 ± 12	77 ± 25	82 ± 29
pH	7.51 ± 0.06	7.32 ± 0.07	7.17 ± 0.05	7.48 ± 0.05	7.29 ± 0.06	7.15 ± 0.05
HCO_3_^−^(mmol/l)	30.5 ± 3.7	32.2 ± 3.9	33.0 ± 3.9	31.1 ± 3.0	33.2 ± 2.8	34.4 ± 3.2
BE(b) (mmol/l)	6.9 ± 3.6	5.1 ± 3.8	3.3 ± 3.6	7.0 ± 3.1	5.6 ± 3.1	4.0 ± 3.5
SpO_2_	97 ± 2	95 ± 4	94 ± 3	96 ± 3	94 ± 4	94 ± 4
MABP (mmHg)	62 ± 9	70 ± 10	75 ± 14	55 ± 8	67 ± 11	74 ± 14
HR (bpm)	139 ± 18	147 ± 28	161 ± 29	140 ± 17	149 ± 25	168 ± 29

### The cerebrocortical microvascular response to graded hypercapnia and NMDA

**Figure 2:**
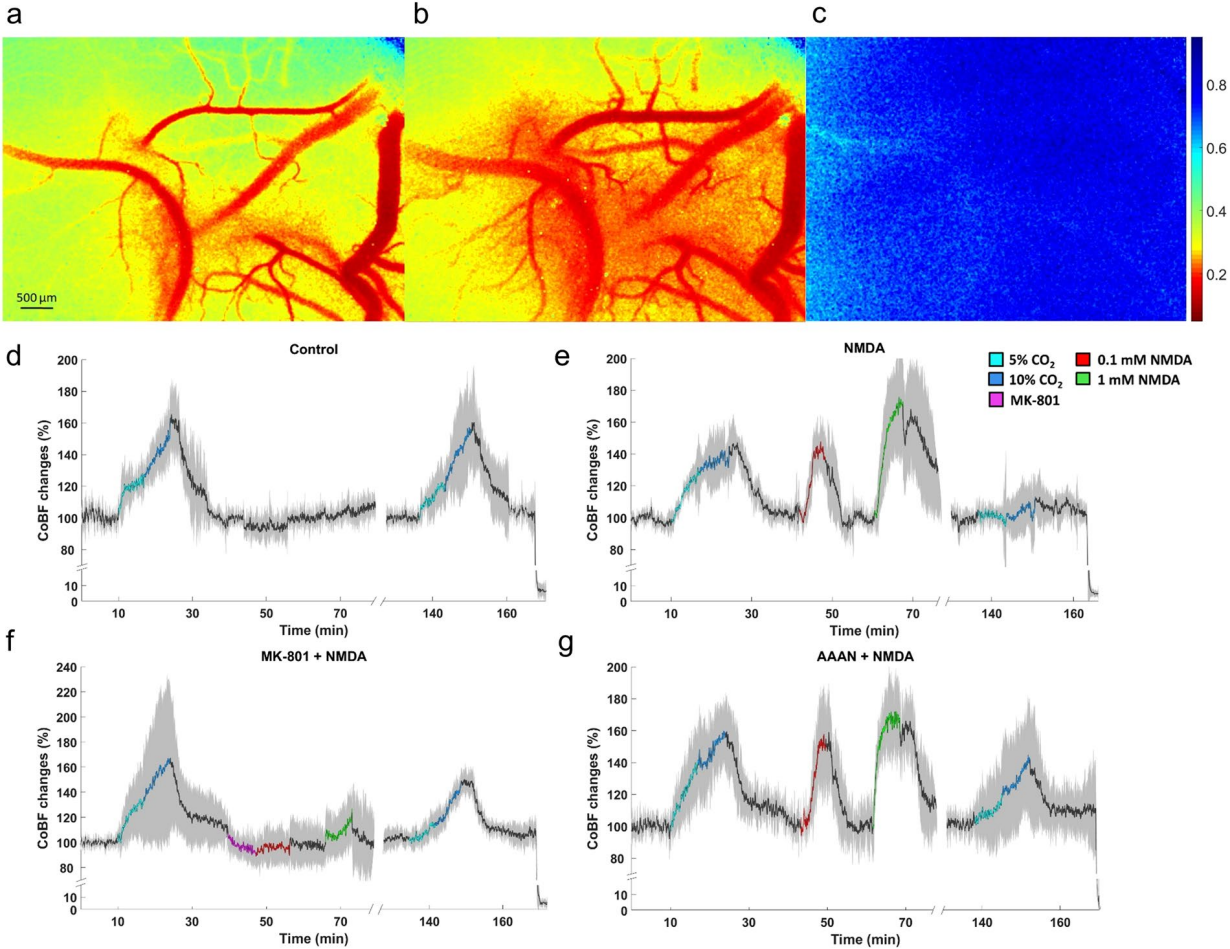
Cortical blood flow (CoBF) changes to graded hypercapnia and NMDA using laser-speckle contrast imaging and analysis. (**a–c**) Representative LSCI contrast images obtained through the closed cranial window (**a–c**) with the corresponding contrast scales. Lower contrast values represent higher flow velocity in the cortical microcirculation. (**a**) baseline condition; (**b**) NMDA (1 mM) showing pial arteriolar vasodilation and increased parenchymal flow as well. (**c**) biological zero after euthanasia characterized by high and stable speckle contrast values marking the disappearance of the perfusion. (**d**–**g**) Summarized recordings of individual experiments, colored/black lines represent the group mean values during/between stimuli, the gray area represent the SD. (**d**) in the control group graded hypercapnia resulted in concentration-dependent repeatable increases in CoBF relative to the baseline. (**e**) Both doses of NMDA reversibly elevated CoBF, however, the CoBF response to graded hypercapnia was virtually absent after NMDA. (**f**) Pre- and co-treatment of MK-801 with NMDA abolished the CoBF response to NMDA and prevented the attenuation of the CoBF response to graded hypercapnia. (**g**) AAAN did not affect the CoBF response to hypercapnia or NMDA, however, at least partially prevented the attenuation of the microvascular response to graded hypercapnia by NMDA.

**Figure 3:**
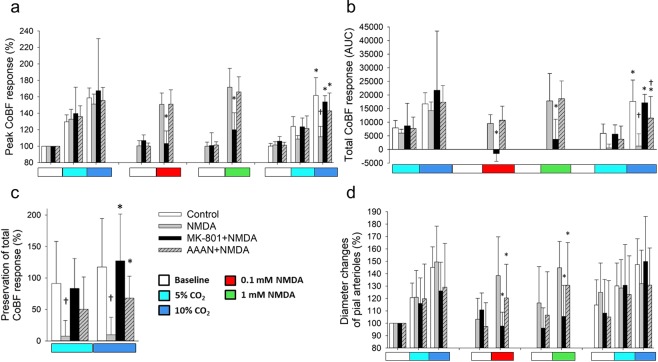
Summary of the cortical microvascular responses to graded hypercapnia and NMDA assessed with laser-speckle contrast imaging and analysis. (**a**) Peak increases in CoBF to graded hypercapnia (n = 6) were significantly blunted after NMDA-treatment (n = 7) alone but not after MK-801 + NMDA coapplication (n = 4) or AAAN pretreatment (n = 7). NMDA-induced peak increases in CoBF were also attenuated by MK-801 (F_group_ = 3.785; η^2^ = 0.066; p = 0.012), but not by AAAN, furthermore AAAN clearly had no effect on the microvascular response to graded hypercapnia. (**b**) determination of the integrated (area under the curve, AUC) CoBF response shows also the severe attenuation of microvascular reactivity to graded hypercapnia in the NMDA-treated animals. Also, the inhibitory effect of MK-801 on NMDA-induced cortical hyperemia is striking (F_group_ = 4.464; η^2^ = 0.411; p = 0.0001). (**c**) Cortical microvascular reactivity to hypercapnia is expressed as the ratio of the total CoBF response to the first and second graded hypercapnia. The response was fully preserved in the control and in the MK-801 + NMDA treated animals, however, virtually abolished in the animals exposed to NMDA alone. Pretreatment with AAAN resulted in a partial preservation of the response (F_group_ = 8.428; η^2^ = 0.441; p = 0.0001). (**d**) Relative (%) changes in pial arteriolar diameters show that pial arteriolar responses to graded hypercapnia were not significantly different among the four groups. However, NMDA-induced pial arteriolar vasodilation was fully prevented by MK-801 and also significantly attenuated by AAAN (F_group_ = 4.741; η^2^ = 0.113; p = 0.004), p < 0.05, * vs. NMDA, † vs. control.

LSCI provided two-dimensional maps of cortical perfusion (Fig. 2a–c) that served to determine changes in parenchymal perfusion (Figs. 2d–g, 3a–c) and pial arteriolar diameters (Fig. 3d).

The first exposure to graded hypercapnia resulted in similar, CO_2_ concentration-dependent increases in CoBF in all groups (Fig. 2d–g), the peak CoBF values (Fig. 3a), the integrated hyperemic CoBF response (Fig. 3b), and the pial arteriolar diameter changes (Fig. 3d) were all similar.

Topical application of 0.1 mM NMDA resulted in a significant increase in CoBF (peak CoBF was 151 ± 14% of baseline) and pial arteriolar diameters (138 ± 31% of baseline) that peaked within 3–4 min (Figs. 2e, 3a,b,d), the elevated CoBF returned to baseline levels after perfusing the cranial window with aCSF. Repeating the stimulation with 1 mM NMDA resulted only in slightly higher elevations both in the peak (172 ± 23%) and the total CoBF responses and the arteriolar diameters (145 ± 20%), and all these changes were reversible upon removal of NMDA. Topical application of the NMDA receptor inhibitor MK-801 did not affect CoBF (Fig. 2f), however, coapplication of MK-801 with either NMDA doses completely abolished the hyperemic and the pial arteriolar response to NMDA (Fig. 3a,b,d). Systemic administration of the selective nNOS inhibitor AAAN did not affect the CoBF response to NMDA (Figs. 2g; 3a,b), however, it caused a significant reduction in the pial arteriolar dilation to NMDA (Fig. 3d).

### NMDA eliminates the hypercapnia-induced cortical microvascular response

In the control group, the CoBF response to the second exposure to graded hypercapnia was virtually identical to the first stimulation (Fig. 2d), the peak and the integrated CoBF values were very similar (Fig. 3a,b), and the cerebrovascular reactivity to either CO_2_ concentration was fully preserved (Fig. 3c). In sharp contrast, cerebrovascular reactivity to the second exposure to graded hypercapnia was abolished in the NMDA-treated group (Figs. 2e, 3). Pre- and coapplication of MK-801 with NMDA prevented the attenuation of the CoBF response to graded hypercapnia (Figs. 2f, 3a,b), cerebrovascular reactivity was preserved (Fig. 3c). In the nNOS inhibitor treated group, the CoBF response to graded hypercapnia was attenuated but not abolished after NMDA (Figs. 2g, 3a,b), thus cerebrovascular reactivity was partially (68 ± 35%) preserved in this group (Fig. 3c).

### LFP changes induced by graded hypercapnia

**Figure 4:**
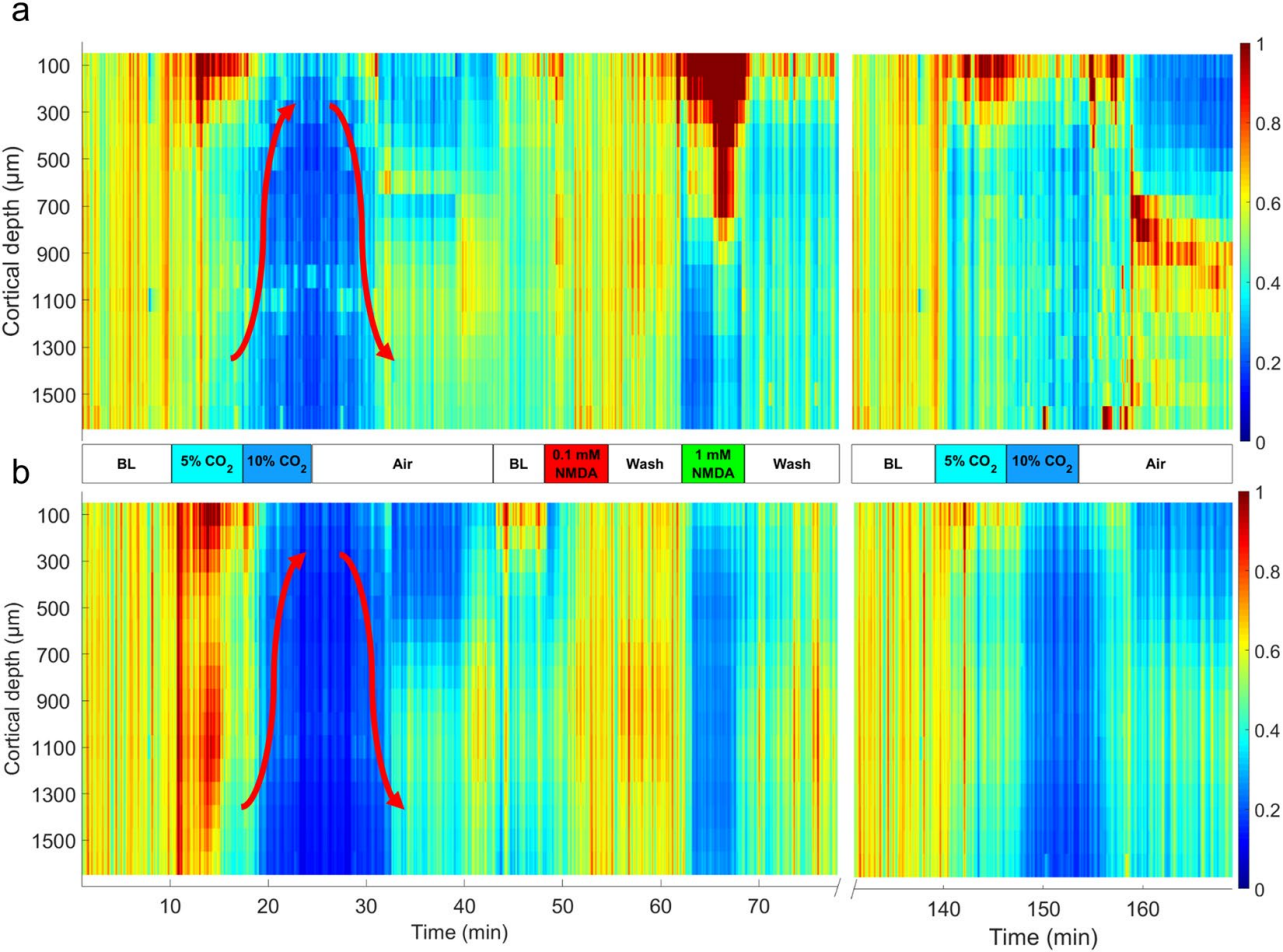
Representative heat map images of (**a**): delta (δ) and (**b**) theta (θ) band power spectral densities (PSDs) obtained from the local field potential recordings with a 16-channel electrode representing 100–1600 μm cortical depths with the corresponding intensity scales (0-1) from an animal of the NMDA-treated group. (**a**) during hypercapnia, 5% CO_2_ tended to increase δ in the superficial cortical layers, but activity diminished after switching to 10% CO_2_ starting in the deep cortical layers gradually shifting upwards (arrow pointing up). After restoration of normocapnia, δ activity was also restored (arrow pointing down). NMDA, especially the higher dose strongly increased δ activity predominantly in the upper cortical layers (F_group_ = 1363.103 (1 mM NMDA); η^2^ = 0.8614; p = 0.00001**). The second stimulation with graded hypercapnia showed a somewhat similar pattern, however, the δ depression during the deeper level of hypercapnia, and also the restoration of activity upon normocapnia was less clear-cut than before NMDA. (**b**) during hypercapnia, 5% CO_2_ resulted in a quite widespread increase in cortical θ activity (F_group_ = 726.632 (5% CO_2_); η^2^ = 0.7623; p = 0.00001**) that was attenuated and reversed to depression after switching to 10% CO_2_ similarly to the pattern observed for δ (arrows). NMDA (1 mM) resulted in an almost complete suppression of θ in all cortical layers. After NMDA, the second graded hypercapnia lacked the θ activation associated with 5% CO_2_. BL: baseline.

Induction of hypercapnia with 5% CO_2_ elicited first increases then decreases in LFP power, especially in the delta (δ) and theta (θ) ranges (Fig. 4a,b). Highest increases in δ were observed at cortical depths 100–400 µm and in θ at 100–600 and at 1000–1200 µm (Table 2). The subsequent reduction in power started in the deeper cortical layers (below 900 µm) gradually shifting upward. Switching to 10% CO_2_ further reduced LFP power both in the δ and θ ranges (Fig. 4). These depressions were largely reversed upon restoration of normocapnia, and the LFPs were not significantly different from the baseline values. LFP power in the alpha (α) and beta (β) ranges was quite small under these experimental conditions and clear hypercapnia-related changes could not be observed (data not shown). LFP changes to graded hypercapnia following the stimulation with NMDA were markedly different, most strikingly the early increases in θ LFP power did not develop (Table 2, Fig. 4b). δ LFP powers were also significantly altered (Table 2), and the pattern of LFP changes appeared to have disorganized.

**Table 2:** Power spectral density changes in the Delta and the Theta band during stimulations with graded hypercapnia and NMDA. Data are expressed as % of baseline (mean ± SD). *p < 0.05, **p < 0.01.

Delta (%)						
Cortical depth (µm)	5% CO_2_	10% CO_2_	0.1 mM NMDA	1 mM NMDA	5% CO_2_	10% CO_2_
100	140.9 ± 14.3*	58.6 ± 17.4	76.2 ± 10.2	252.1 ± 54.3**	51.2 ± 5.1	50.5 ± 12.3
200	130.4 ± 9.2*	20 ± 3.1	44.7 ± 5.1	243.6 ± 49.2**	46.0 ± 8.2	22.3 ± 2.0
300	123.9 ± 23.6*	18.7 ± 3.1	46.8 ± 4.1	187.0 ± 14.3**	45.3 ± 10.3	39.5 ± 11.3
400	127.9 ± 28.7*	46.5 ± 8.2	113.4 ± 11.3	305.0 ± 78.9**	78.5 ± 12.3	68.2 ± 23.6
500	79.8 ± 13.3	54.3 ± 8.2	92.5 ± 5.1	191.1 ± 14.3**	66.6 ± 11.3	59.6 ± 18.4
600	71.1 ± 7.2	44.1 ± 12.3	90.3 ± 7.2	197.7 ± 15.4**	60.8 ± 16.4	50.3 ± 25.6
700	38.0 ± 3.1	24.1 ± 7.2	49.9 ± 5.1	160.6 ± 11.3**	24.4 ± 2.0	34.8 ± 8.2
800	73.4 ± 14.3	60.4 ± 29.7	113.0 ± 10.2	140.9 ± 18.4*	82.1 ± 8.2	77.0 ± 5.1
900	52.1 ± 5.1	46.1 ± 10.2	67.3 ± 3.1	66.7 ± 10.2	97.9 ± 21.5	65.6 ± 13.3
1000	29.7 ± 4.1	26.6 ± 6.1	37.7 ± 1.0	54.2 ± 7.2	31.6 ± 8.2	31.0 ± 6.1
1100	83.0 ± 7.2	39.3 ± 10.2	103.8 ± 5.1	74.0 ± 19.5	61.7 ± 9.2	53.3 ± 10.2
1200	40.8 ± 6.1	31.5 ± 10.2	33.7 ± 3.1	40.7 ± 7.2	27.2 ± 4.1	52.1 ± 14.3
1300	65.7 ± 5.1	40.6 ± 8.2	56.4 ± 2.0	75.7 ± 14.3	42.3 ± 4.1	50.6 ± 7.2
1400	55.3 ± 6.1	41 ± 7.2	45.1 ± 2.0	80.8 ± 21.5	38.4 ± 3.1	65.3 ± 12.3
1500	52.7 ± 3.1	27.7 ± 2.0	60.1 ± 3.1	58.4 ± 9.2	45.8 ± 8.2	36.3 ± 8.2
1600	64.2 ± 5.1	35.2 ± 4.1	61.6 ± 7.2	84.0 ± 14.3	40.1 ± 4.1	34.5 ± 7.2
**Theta (%)**						
100	184.4 ± 14.3**	32.1 ± 5.1	61.9 ± 3.1	59.0 ± 11.3	43.2 ± 4.1	24.7 ± 3.1
200	174.2 ± 13.3**	22.7 ± 2.0	80.5 ± 6.1	38.1 ± 9.2	42.4 ± 3.1	21.6 ± 3.1
300	163.7 ± 14.3**	19.1 ± 1.0	59.0 ± 4.1	51.3 ± 8.2	37.9 ± 3.1	31.5 ± 4.1
400	160.1 ± 18.4**	24.5 ± 3.1	76.8 ± 6.1	57.7 ± 11.3	42.2 ± 4.1	30.6 ± 4.1
500	157.5 ± 14.3**	30.3 ± 5.1	51.2 ± 3.1	42.5 ± 8.2	31.3 ± 3.1	29.1 ± 6.1
600	165.4 ± 18.4**	21.7 ± 2.0	108.9 ± 9.2	61.7 ± 11.3	42.4 ± 4.1	31.8 ± 3.1
700	105.0 ± 15.4	58.5 ± 12.3	77.7 ± 6.1	67.1 ± 11.3	41.1 ± 3.1	38.4 ± 4.1
800	105.1 ± 12.3	19.9 ± 1.0	81.2 ± 6.1	56.8 ± 10.2	49.1 ± 7.2	40.9 ± 7.2
900	114.1 ± 11.3	29.9 ± 3.1	96.9 ± 7.2	57.4 ± 9.2	65.4 ± 4.1	41.9 ± 5.1
1000	167.5 ± 11.3**	22.3 ± 2.0	91.9 ± 6.1	63.9 ± 10.2	53.1 ± 4.1	44.5 ± 8.2
1100	157.7 ± 12.3**	19.4 ± 2.0	86.7 ± 7.2	59.1 ± 9.2	55.6 ± 3.1	50.4 ± 8.2
1200	154.0 ± 12.3**	24.5 ± 2.0	100.8 ± 7.2	55.3 ± 9.2	56.9 ± 3.1	44.0 ± 7.2
1300	114.5 ± 15.4	22.3 ± 2.0	98.1 ± 6.1	54.0 ± 7.2	64.3 ± 4.1	61.9 ± 1.0
1400	109.5 ± 17.4	17.7 ± 1.0	83.9 ± 5.1	52.0 ± 7.2	59.3 ± 3.1	40.9 ± 6.1
1500	101.5 ± 16.4	19 ± 2.0	82.0 ± 6.1	45.8 ± 7.2	60.4 ± 4.1	32.4 ± 4.1
1600	104.1 ± 16.4	32.7 ± 3.1	71.3 ± 5.1	62.5 ± 7.2	56.2 ± 3.1	48.9 ± 6.1

### NMDA evokes delta (δ) oscillation

**Figure 5:**
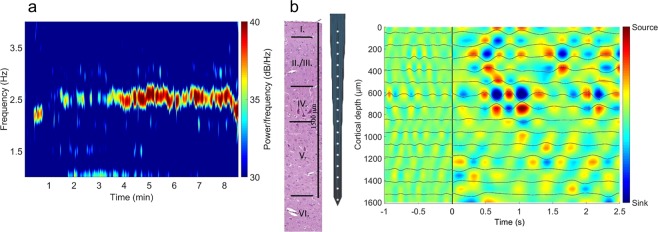
NMDA evokes delta (δ) oscillation in the upper cortical layers. (**a**) Representative δ band spectrogram during NMDA (1 mM) stimulation recorded at 600 µm under the cortical surface. The heat map shows the appearance of a 2.5 Hz frequency oscillation. (**b**) Average current source density (CSD) map of the δ oscillations observed during NMDA (1 mM) stimulation with the δ-filtered LFP superimposed onto the image. The multi-channel electrode (http://neuronexus.com/electrode-array/a1x16–10mm-100-177/) and an H/E-stained section of the piglet cortex are also shown for orientation. CSD analysis show that the oscillation originates chiefly in layers III/IV where the largest amplitude currents appear.

NMDA (1 mM) selectively increased δ LFP power only in the upper layers (100–700 µm; Fig. 4a; Table 1) however, activity in the θ range were simultaneously suppressed (Fig. 4b). Similar suppression was observed in the α and β ranges as well (e.g. at 600 µm cortical depth reductions were 58.8 ± 1.2 and 44.9 ± 1.7% of baseline, respectively). This characteristic increase in the δ was identified as a ~2.5 Hz δ oscillation) down to 600 µm (Fig. 5a). CSD analysis identifying the contributing sinks and sources of cortical extracellular currents showed that NMDA (1 mM) altered significantly the size of the sinks and sources (Fig. 5b) causing the activation first in layer I. then layer II/III and IV. This NMDA-induced activation was δ band-limited.

### Neuronal spiking in response to graded hypercapnia and NMDA

**Figure 6:**
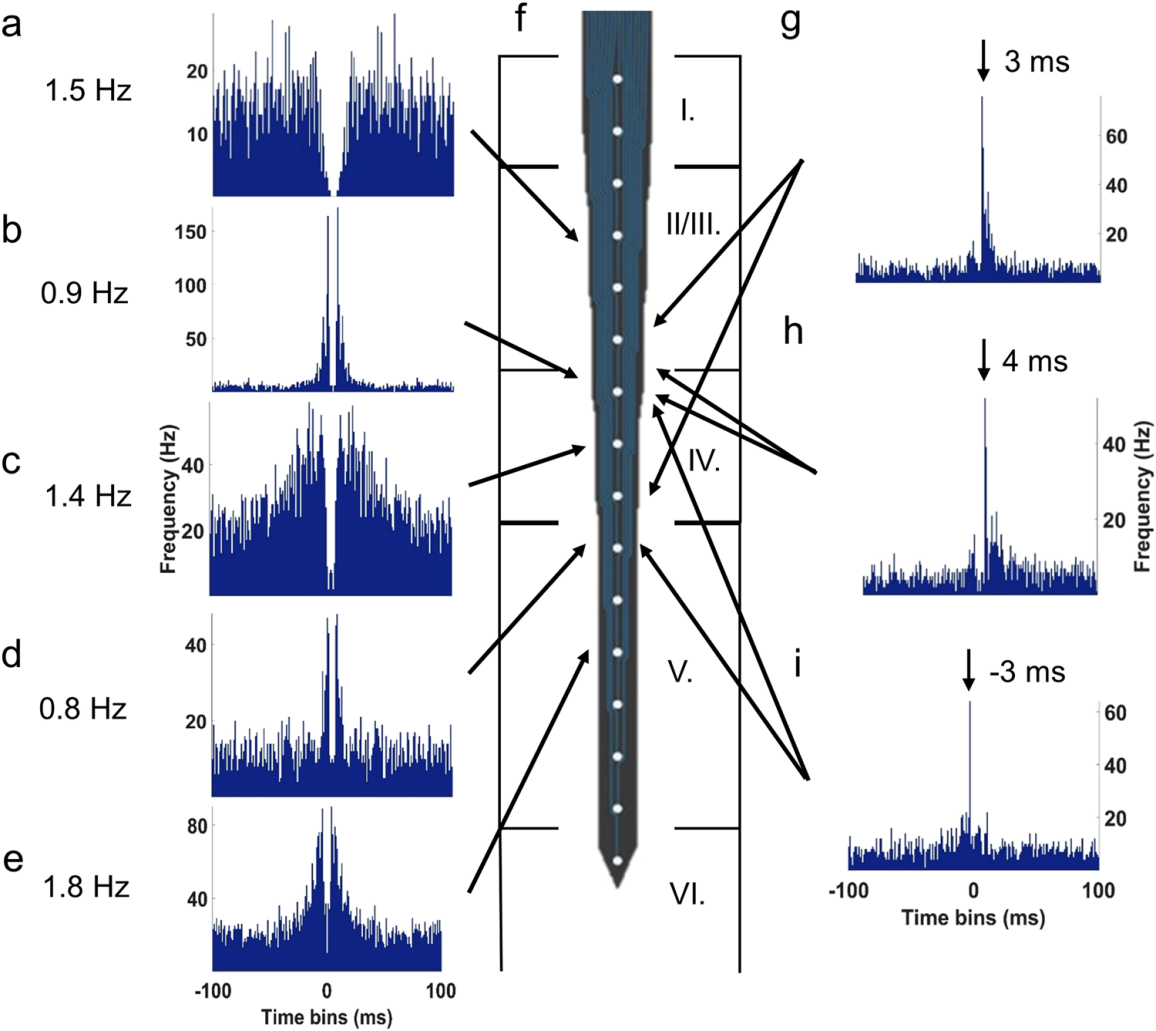
Representative auto- and cross-correlograms (ACGs and CCGs, respectively) of recorded cortical neurons and their connections with their highest firing rates. (**a**) Layer II/III. interneuron. (**b**) Layer IV. bursting pyramidal cell. (**c**) Layer IV. regular spiking pyramidal cell. (**d**,**e**) Layer V. bursting pyramidal cells. (**f**) Representative image of the recording electrode (http://neuronexus.com/electrode-array/a1x16-10mm-100-177/) with its position in the parietal cortex. Excitatory synapses between a (**g**) bursting and a regular spiking, (**h**) interneuron and bursting pyramidal cell, (**i**) two bursting pyramidal cells.

All recorded neurons (n = 149; total spike count = 152,089) fired with low frequency (~0.3–3 Hz, Fig. 6a–e). The ACGs identified major neuronal types of the parietal cortex such as interneurons (Fig. 6a), bursting (Fig. 6bde) and regular spiking pyramidal cells (Fig. 6c). CCGs helped to identify the most characteristic cell connections that were typically excitatory with 3–4 ms latency (Fig. 6g–i). We observed only a few inhibitory connections or reciprocal excitation/inhibition between the cells (Fig. 6h).

Graded hypercapnia and NMDA increased spiking activity mainly in the II/III. and IV. layers down to 900 µm. Increased firing allowed more precise identification of interneuronal connections, from the total 164 connections 71% have been associated with layer II/III including intralaminar connections as well. Under the first hypercapnia the recorded spike counts were much higher compared to baseline condition (5% CO_2_: 36,245 spikes, 146/149 active cells; 10% CO_2_: 24,043 spikes, 142/149 active cells). In the subsequent normocapnic period the spiking activity has returned almost to the baseline activity (19,443 spikes, 133/149 active cells). NMDA (1 mM) suppressed neuronal activity in ~60% of neurons, the number of active cells dropped to 73/149, but the remaining ~40% of neurons were excited that total spike count remained similar to the baseline (15,400 spikes). During the second hypercapnia, few cells remained responsive CO_2_ (5% CO_2_: 41/149 active cells, 8985 spikes; 10% CO_2_: 17/149 active cells, 5130 spikes).

## Discussion

The major novel findings of the present study are the following: (1) Graded hypercapnia elicits repeatable, concentration-dependent increases in CoBF that was attenuated by topical application of NMDA. (2) The attenuation of the hemodynamic response to hypercapnia after NMDA was prevented fully by MK-801 and partially by AAAN indicating an exclusive role for the NMDA receptor and a significant role for nNOS activation in mediating this response. (3) The attenuated cerebrovascular response after NMDA was associated with an altered electrophysiological response to hypercapnia, more specifically, the increased PSD in the δ and θ bands of the LFP was greatly attenuated. (4) NMDA, however, was shown to elicit strong, ~2.5 Hz delta oscillations peaking at 5–700 μm from the cortical surface indicating the major cortical targets of topically applied NMDA.

### The cortical microvascular response to NMDA

The newborn pig is an established translational large animal model of the term neonate, thus the study of cerebrocortical microvascular reactivity to various stimuli as well as its alterations following hypoxic/ischemic stress in this model are of great interest to investigative neonatology^22,23^. Accordingly, the closed cranial window/intravital microscopy technique has been used extensively to study the cerebrocortical microcirculation. NMDA-induced pial arteriolar vasodilation was indeed first described in this species^24^, later also confirmed in other species studied such as in rabbits^25^ and rats^26^. Based on its sensitivity to tetrodotoxin^27^ and nNOS inhibitors^28,29^, the mechanism of NMDA-induced pial arteriolar dilation has been accepted to be mediated by neuronal NO production in piglets and in most other experimental models^30^. The present study simultaneously confirms previous experimental findings but conflicts with the conclusions drawn in previous studies. In the present study nNOS inhibition by AAAN (0.4 mg/kg) resulted in a similar attenuation (~30–40%) of pial arteriolar dilation to NMDA to that found by Bari *et al*. using the nNOS inhibitor 7-nitroindazole (50 mg/kg)^29^. However, we now demonstrated that the attenuated arteriolar vasodilation to NMDA did not coincide with attenuated blood flow response in the underlying parenchyma, indeed the parenchymal flow response was virtually unchanged. We believe that the LSCI used in the present study to assess the cortical microvasculature gives a good estimate of the NMDA-induced microvascular response as our current results showing ~72% increase in CoBF to 1 mM NMDA are in complete agreement with our previous study displaying virtually identical increases in CoBF to this dose of NMDA using laser-Doppler flowmetry^6^. Our results suggest that dilation of intraparenchymal arterioles plays a more decisive role in determining the CoBF response and can compensate for the somewhat smaller pial arteriolar dilation in response to NMDA when nNOS activity is compromised. This finding is in contrast with data obtained in the adult rat, where also the CoBF response to NMDA applied was demonstrated to be critically dependent on nNOS presence and activity^31^, and this difference may well represent a species/age-dependent difference. Clearly, additional vasodilatory mechanisms acting perhaps predominantly on intraparenchymal vessels must play a role in mediating the observed increases in CoBF to NMDA in the piglet that can be subject to further research. One such mediator could be adenosine, as adenosine was proposed beside NO to contribute to the NMDA-induced CoBF response in the rat cerebral cortex based on microdialysis data^32^. In the piglet cortex, exogenous adenosine appeared to reduce the pial arteriolar response to NMDA^33^, this latter finding is conceivable presuming the adenosine may have had a predominant action of intraparenchymal arterioles that based on the present study can have responses largely independent of the pial vessels.

### The cortical neuronal response to NMDA

The cortical microvascular response to NMDA in piglets is thought to be mediated by exclusive activation of neuronal NMDA receptors based on two lines of evidence, isolated piglet cortical vessels do not respond to NMDA^34^, and functional NMDA receptors are not expressed in cerebrovascular endothelial cells or microvessels isolated either from rats, humans or piglets^35,36^. However, recent work suggests that in mice abluminally localized endothelial NMDA receptors can contribute to functional hyperemia^37,38^. Unlike the painstakingly analyzed pial arteriolar response to NMDA, the changes in neuronal activity in response to topically applied NMDA were hardly tackled. Topically applied NMDA was demonstrated to elicit an SD characterized by the signature SD-related DC potential shift simultaneously with pial arteriolar changes in adult mice, and the NMDA effect was found concentration-independently linked to the evoked SD^5^. In a subsequent adult rat study, topical NMDA was indeed found to also elicit an SD, but the CoBF response after the SD-related hemodynamic response demonstrated additionally also an NMDA-dose dependent component^6^. The same experimental approach in piglets found only a dose-dependent CoBF response without any confounding SDs^6^. Immunohistochemistry studies in the piglet parietal cortex identified layers II/III as the major site of nNOS-immunoreactive neurons and as these superficial cortical layers were also rich in NMDA-receptor immunoreactive nerve cells^33^, they were accordingly assumed to be responsible for the observed effects of NMDA. Our present study is essentially the first that studied the electrophysiological response simultaneously in all cortical layers to topically applied NMDA. Our present findings identified not only the most superficial, but also much deeper cortical structures to be affected by topical NMDA. Thus, the NMDA-induced intense 2.5 Hz δ-oscillation have been found most prominent at 6–800 μm from the cortical surface suppressing LFP power in virtually all other frequency bands. NMDA also had opposing effects on neuronal firing, it suppressed or stimulated spiking in different neuronal populations. We found that after NMDA, the LFP powers were restored to baseline levels, and also no abnormal spikes were recorded during/after NMDA application suggesting that significant excitotoxic lesion to the cortex did not occur during NMDA stimulation. This notion is in accordance with previous findings showing that the neurovascular response to topical NMDA is preserved in piglets (four applications in a 5 hours period)^39^. These results correspond well both with the previous observations of SD-triggering and also with the tetrodotoxin-sensitive actions of topical NMDA application.

### NMDA triggers SD-like suppression of the microvascular response to hypercapnia

The cortical microvascular response to graded hypercapnia has been extensively studied in the piglet, but few experiments assessed pial arteriolar diameter and CoBF changes simultaneously^40^. In this study^40^, both responses were unaltered by the general NOS inhibitor Nω-nitro-L-arginine methyl ester, indicating that NO plays minor role in the mediation of this response in the newborn pig. This NO-independence appears to be age-restricted in the pig, the pial arteriolar response to hypercapnia has also been shown to be NO-independent in newborns, but to be partially NO-dependent in juvenile (3–4 months old) pigs^41,42^. In the rat, the soluble guanylate cyclase inhibitor 1 H-[1,2,4]oxadiazolo[4,4,-a]quinoxalin-1-one (ODQ) had also no effect on the response to hypercapnia but to NMDA^43^. However, a number of rat studies emphasize the role of nNOS-derived NO in the CoBF response to hypercapnia^44,45^. In accordance with previous newborn pig studies, the nNOS inhibitor AAAN had no effect on the cortical microvascular response to graded hypercapnia in the present study.

The pial arteriolar response to graded hypercapnia has been known to be vulnerable to hypoxic/ischemic stress^46^, endothelial injury^47^, or seizures^48^ making the response a good indicator of neurovascular unit dysfunction. SDs are also known to attenuate the microvascular response to hypercapnia in adult brains^2–4^. In the piglet cortex *bona fide* SDs of course could not be generated, however, an artificially induced 3-min long cortical depolarization (elicited with topical KCl and confirmed with DC recording) did not affect the microvascular response to hypercapnia and other assessed stimuli^49^. In contrast, in the present study, NMDA attenuated the response to graded hypercapnia in the newborn cerebral cortex that is similar to that observed after SD in adult cortex. The microvascular alteration appears to be independent of the direct hemodynamic effect of NMDA-receptor activation but its mechanism appears to involve nNOS activity. However, generalization of our findings to the adult cerebral cortex is prevented by the apparent limitations of developmental differences that actually prevent the triggering of SD in the neonatal brain.

In the present study, we just started to decipher the connections between the well-known cerebrovascular effects of hypercapnia and NMDA and the virtually uncharted neuronal effects of these stimuli in the cerebral cortex of the newborn pig. Using multi-channel silicone probes to study LFP and unit activity changes are widely used in the literature^50,51^, although for instance interpretation of LFP data is difficult due to the many sources contributing to the mixed signal^52,53^. There is very little information available about the LFP^54^ or the unit activity^55^ of the adult pig cerebral cortex let alone of the newborn piglet. Our current findings show layer-specific and concentration-dependent effects of hypercapnia on both the LFP and unit activity that are clearly altered after NMDA. Presently, we cannot make causative statements whether alterations in the neuronal response trigger the observed changes in microvascular reactivity or perhaps vice versa, however, our results are strongly indicating that the mechanism of NMDA-induced attenuation of the microvascular response is not likely to be limited to the cerebral vasculature. NMDA has been also shown to suppress all frequency bands of the LFP (similar to an SD in adult or old rats^56–58^), but it also triggered a 2.5 Hz delta-oscillation. The origin of the arising delta-oscillation is still not clear, however it has been described earlier that these oscillations can be evoked by topical stimulations and different cell types can generate abnormal oscillations in the cortex as well^59–62^. Furthermore, spiking was also suppressed in some, but triggered in other units during NMDA. Again, we cannot determine the significance of these observations in the attenuation of microvascular response to hypercapnia, however, we can hypothesize that different components of the electrophysiological response to NMDA could be responsible for the attenuation of microvascular reactivity to hypercapnia and for the developing marked CoBF response. Clearly, further studies are warranted to identify the neuronal-vascular mechanisms selectively responsible for either effect of topically applied NMDA.

In conclusion, NMDA triggers microvascular dysfunction in the piglet cerebral cortex similar to but not confounded by SD. Understanding the mechanism of this novel observation may help to elucidate the deleterious effects of SD on the neurovascular unit.

## Data Availability

The datasets generated during the current study are available from the corresponding author on reasonable request.
